# Mudskippers and Their Genetic Adaptations to an Amphibious Lifestyle

**DOI:** 10.3390/ani8020024

**Published:** 2018-02-07

**Authors:** Xinxin You, Min Sun, Jia Li, Chao Bian, Jieming Chen, Yunhai Yi, Hui Yu, Qiong Shi

**Affiliations:** 1 Shenzhen Key Lab of Marine Genomics, Guangdong Provincial Key Lab of Molecular Breeding in Marine Economic Animals, BGI Academy of Marine Sciences, BGI Marine, BGI, Shenzhen 518083, China; youxinxin@genomics.cn (X.Y.); sunmin_wolf@163.com (M.S.); lijia1@genomics.cn (J.L.); bianchao@genomics.cn (C.B.); chenjieming@genomics.cn (J.C.); yiyunhai@genomics.cn (Y.Y.);yuhui@genomics.cn (H.Y.); 2 BGI Education Center, University of Chinese Academy of Sciences, Shenzhen 518063, China; 3 BGI-Zhenjiang Institute of Hydrobiology, BGI Marine, BGI, Zhenjiang 212000, China; 4 Laboratory of Aquatic Genomics, College of Life Sciences and Oceanography, Shenzhen University, Shenzhen 518060, China

**Keywords:** mudskipper, amphibious lifestyle, genome, transcriptome, terrestrial adaptation

## Abstract

**Simple Summary:**

Mudskippers are an interesting group of goggle-eyed amphibious fish that can live both in water and on land. They are a useful model for obtaining insights into the genetic mechanisms underlying the terrestrial adaptations of amphibious fish. This review summarizes the morphological and physiological modifications of representative mudskippers, and focuses on the recent advancement of genomic studies on their genetic adaptations to the amphibious lifestyle.

**Abstract:**

Mudskippers are the largest group of amphibious teleost fish that are uniquely adapted to live on mudflats. During their successful transition from aqueous life to terrestrial living, these fish have evolved morphological and physiological modifications of aerial vision and olfaction, higher ammonia tolerance, aerial respiration, improved immunological defense against terrestrial pathogens, and terrestrial locomotion using protruded pectoral fins. Comparative genomic and transcriptomic data have been accumulated and analyzed for understanding molecular mechanisms of the terrestrial adaptations. Our current review provides a general introduction to mudskippers and recent research advances of their genetic adaptations to the amphibious lifestyle, which will be helpful for understanding the evolutionary transition of vertebrates from water to land. Our insights into the genomes and transcriptomes will also support molecular breeding, functional identification, and natural compound screening.

## 1. Introduction

**Figure 1:**
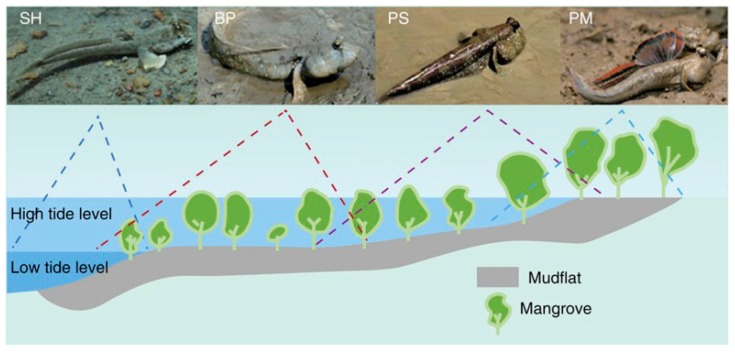
Representative mudskippers and their habitats [5]. *Scartelaos histophorus* (SH) and *Boleophthalmus pectinirostris* (BP) are predominantly water-dwelling, whereas *Periophthalmodon schlosseri* (PS) and *Periophthalmus magnuspinnatus* (PM) spend extended periods of time on land.

Amphibious fish spend periods of time out of water, in or above the ground surface, as normal parts of their life histories [1]. Mudskippers (Figure 1) are a major group of amphibious fish with an enormous potential for theoretical research into critical adaptations to facilitate the evolution from an aqueous to a terrestrial lifestyle. They are divided into four main genera, including *Boleophthalmus*, *Periophthalmodon*, *Periophthalmus,* and *Scartelaos* [2]. Mudskippers have developed morphological and physiological terrestrial adaptations to match their unique lifestyle, such as modification of aerial vision, higher ammonia tolerance, and terrestrial locomotion using protruded pectoral fins [3,4,5].

**Figure 2:**
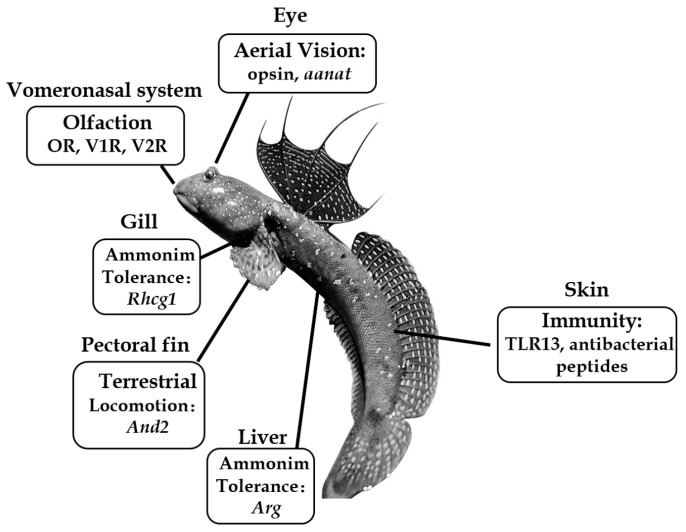
Summary of important genes related to the amphibious lifestyle of mudskippers. The blue-spotted mudskipper (*B. pectinirostris*, BP) is jumping on the mudflat. Abbreviations: *aanat*, aralkylamine *N*-acetyltransferase; *And2*, actinodin 2; *Arg*, arginase; *Rhcg1*, Rhesus C glycoprotein 1; *TLR13*, Toll-like receptor 13.

By presenting the first genome sequences of amphibious fish [5], we provide a new model for understanding the adaptive evolution of animals from water to land. In the genome report, we sequenced four representative species of mudskippers (Figure 1), including *Boleophthalmus pectinirostris* (BP or blue-spotted mudskipper), *Scartelaos histophorus* (SH or blue mudskipper), *Periophthalmodon schlosseri* (PS or giant mudskipper), and *Periophthalmus magnuspinnatus* (PM or giant-fin mudskipper). The four mudskippers form a monophyletic clade that diverged from the other teleosts ~140 million years ago [5]. BP and SH form one sister group, and are predominantly aqueous and spend less time out of water, whereas PS and PM constitute another sister group, and are primarily terrestrial and spend extended periods of time on land. Interestingly, the genome sizes decrease in the following order: BP > SH > PM > PS, which may be associated with their terrestrial affinity but unrelated to their body size (PS > BP > SH > PM). Comparative genomic analyses were carried out to gain insights into the fundamental genetic basis of terrestrial adaptation in mudskippers. Since then, more and more knowledge about genetic adaptations to the amphibious lifestyle has been accumulated (Figure 2).

Mudskippers spend part of their lives out of water to feed, mate, and avoid capture by terrestrial predators. Therefore, they have developed behavioral and physiological specializations for adaptation to the amphibious life. The most significant morphological and functional modifications (Figure 1) are the trend to possess close-set and moveable protuberant eyes set high on the head for escape from predators on land, and the eyes are structural adapted for accurate vision in both air and water [6]; higher ammonia tolerance allow the fish to live in the high concentration of NH_3_/NH_4_^+^ within the intertidal zone [7,8,9]; stronger pectoral fins help mudskippers crawl and jump in the mudflat [10]; higher disease resistance enables mudskippers to inhabit both aqueous and terrestrial environments [5]; and specialized skin retains more water for them to stay on land for a longer period [6].

The main objective of this review is to summarize the current knowledge of genetic adaptations to the amphibious lifestyle of mudskippers, such as aerial vision and olfaction modifications, ammonia tolerance mechanisms, terrestrial locomotion, immunological difference, and air exposure response.

## 2. Modification of Aerial Vision

Fully aqueous teleost fish are likely to have myopic vision in air; however, mudskippers seem to possess a good aerial vision (Figure 1; [11]). To investigate this phenomenon, genomic analysis was applied to prove related modification of their visional changes to avoid terrestrial predators. We discussed adaptation for their aerial vision via loss of aralkylamine *N*-acetyltransferase 1a (*aanat1a*), and modifications of wavelength-sensitive-related genes [5,12,13].

Photoreceptor cells (in retinae) and pinealocytes (in pineal gland) are active for light detection in amphibians, reptiles, and fish [6]. Typically, there are five types of visual opsins in retina of vertebrates, including LWS (long wavelength-sensitive), SWS1 (short wavelength-sensitive 1), SWS2 (short wavelength-sensitive 2), RH1 (rhodopsin 1), and RH2 (rhodopsin 2, green-sensitive). However, certain vision-related genes have been adaptively lost or mutated in mudskippers [5]. For example, SWS1s are often used for recognition of ultraviolet vision. However, the SWS1s of many vertebrates (i.e., human, cow, chicken, etc.) have shifted more towards violet light rather than ultraviolet light, thus minimizing retinal damage from ultraviolet light [14,15]. Mudskippers may overcome the increased exposure to ultraviolet light by making SWS1 less effective to prevent UV damage, and by allowing it to be lost from the genomes of both BP and PM. We also found that both BP and PM have a broader range of color sensitivities between LWS1 and LWS2 than other teleost [5]. Therefore, we assumed that the two LWS opsins in mudskippers are adaptations for the improvement of color vision on land [5,16].

**Figure 3:**
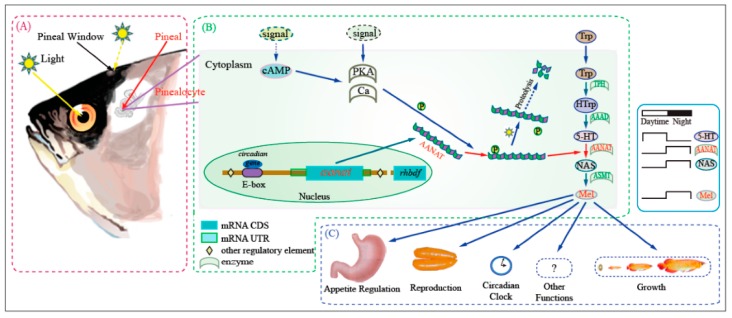
Outline of melatonin biosynthesis in the fish pineal gland [12]. (**A**) Photoreceptive and transmission system; (**B**) Melatonin synthesis pathway; (**C**) Biological functions of melatonin. Aralkylamine *N*-acetyltransferase (AANAT, shown highlighted in the center of the figure), is the key to melatonin biosynthesis in vertebrates, being upregulated and downregulated on a circadian rhythm with other neuromodulators. AANAT exists in tetrapods as a single gene, whereas there are three genes in teleosts (AANAT1a, AANAT1b, and AANAT2). BP contains all the three AANATs, whereas PM has lost AANAT1a [4]. Abbreviations: AAAD, aromatic-l-amino-acid decarboxylase; ASMT, acetylserotonin-*O*-methyltransferase; Ca, calcium; cAMP, cyclic adenosine monophosphate; PKA, protein kinase A; HTrp, 5-hydroxytryptophan; 5-HT, 5-hydroxytryptamine; NAS, *N*-acetylserotonin; MEL, melatonin; Trp, tryptophan; TPH, tryptophan hydroxylase.

Aralkylamine *N*-acetyltransferase (AANAT), the most important enzyme for the daily cycle of melatonin biosynthesis (Figure 3), exists in tetrapods (a single *aanat* gene) and teleosts (*aanat1a*, *aanat1b* and *aanat2*). BP contains all the three AANATs, whereas PM lost AANAT1a [5]. The recently identified function of AANAT1a in retina is dopamine acetylation, which has been proposed to reduce the retinal dopamine levels and lead to myopia development [17,18]. This gene loss therefore can increase the dopamine concentration in retina so as to change the shortsightedness condition and help PM live on land for an extended period [5,12,16].

## 3. Higher Ammonia Tolerance

Mudskippers are remarkably high ammonia-tolerant fish and possess various strategies to ameliorate ammonia toxicity during exposure to environmental ammonia. Ornithine-urea cycle is used to produce urea as a way to detoxify ammonia in tetrapods [8]; however, mudskippers could survive both in water and on land by a combination of active NH_3_/NH_4_^+^ excretion and low membrane permeability for ammonia [5,19], and employ part of the amino acid metabolism under the ammonia-rich condition [20]. We elaborate these two mechanisms as follows.

### 3.1. Ammonia Excretion Pathway

For the mudskipper PS, Wilson et al. [21] provided evidence for the hypothesis that ion-transport proteins in gill mitochondria-rich (MR) cells contribute to active ammonia (NH_4_^+^) excretion. We also found that PM has acquired a greater capacity to tolerate higher environmental ammonia than BP, and provided transcriptomic supports through profiling the gill and liver transcriptomes of both BP and PM [20].

**Figure 4:**
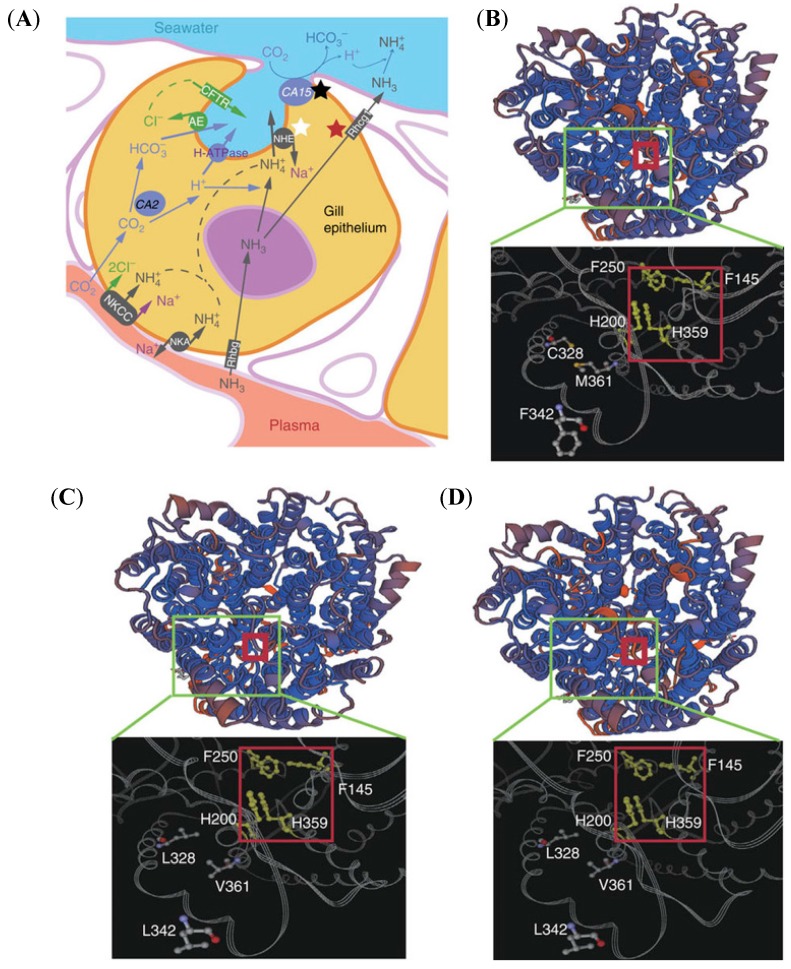
An overview of ammonia excretion pathways in the mudskipper gills [5]. (**A**) An overview of ammonia excretion pathways in the gills illustrates the differential ammonia excretion in mudskippers. The core pathway comprises Na^+^-K^+^-Cl^−^ co-transporter (NKCC), Na^+^K^+^-ATPase (NKA), carbonic anhydrase (CA), cystic fibrosis transmembrane conductance regulator (CFTR), Na^+^/H^+^ exchanger (NHE) 3, H^+^-ATPase-V-type-B-subunit (H-ATPase), anion exchanger (AE), and glycosylated Rhesus protein b (Rhbg) and c (Rhcg1 and Rhcg2). The black star represents genes with positive selection in both BP and PM, whereas the white and red stars indicate genes that are positively selected specifically in BP and PM, respectively. (**B**–**D**) Three-dimensional views of Rhcg1 proteins (predicted and copied from Protein Data Bank) in BP (**B**), PM (**C**), and PS (**D**) highlight several PM- and PS-specific amino-acid substitutions. The red squares indicate the central pore of the channel for transporting NH_3_, which includes the conserved Phe-Gate (F145, F250) and Twin-His (H200, H359). Three genetic variations around the central pore, Leu328Cys, Leu342Phe, and Val361Met in PM and PS, may be related to a more-efficient NH_3_ diffusion system in PM and PS suited for a land-dominant lifestyle.

In the ammonia excretion process in gill, several representative genes are commonly increased in transcription (Figure 4A). BP excretes NH_4_^+^ to face the high ammonia condition via NH_4_^+^ transporting and H^+^ excreting related carbonic anhydrase (*CA15*) and Na^+^/H^+^ exchanger (*NHE3*) [5], which are up-regulated at levels greater than those found in PM.

PM lives mostly out of water, and its excretion of NH_3_ could be volatilized without moisture removal and acid damage to fish skin. Under the high ammonia stress, the transcription levels of Na^+^/K^+^(NH_4_^+^)-ATPase (*Nka*), Na^+^/K^+^(NH_4_^+^)/2Cl^−^ cotransporter 1 (*Nkcc1*), *CA15,* and NH_3_ transporting channel related Rhesus glycoprotein type c (*Rhcg1*) are up-regulated [5]. We also observed that there are more hydrophobic amino acid residues in the functional center of Rhcg1 (Figure 4B–D) in PM that could excrete the NH_3_ more effectively than BP. Interestingly, our genomic data proposed a positive selection of the *CA15* gene in both BP and PM, whereas *NHE* and *Rhcg1* genes are positively selected specifically in BP and PM, respectively.

### 3.2. Amino Acid Metabolism for Ammonia Tolerance

Ammonia is mainly generated through amino acid catabolism that occurs in the liver mitochondria of ammonotelic fish. Mudskippers could decrease the production rate of ammonia from amino acid catabolism to slow down the buildup of internal ammonia under ammonia exposure [22]. Interestingly, partial amino acid catabolism leads to the formation of alanine in mudskippers and facilitates the use of amino acids as an energy source during locomotor activity on land [23].

We provided molecular evidence for mudskippers’ adoption of partial amino acid catabolism to decrease the production of endogenous ammonia under high environmental ammonia loading [20]. The critical pathway could be summarized as follows: some amino acids (arginine, glutamine, histidine, and proline) are decomposed to glutamic acid; meanwhile, malic acid in tricarboxylic (TCA) cycle is transformed into pyruvic acid by malic enzyme. Subsequently, pyruvic acid accepts the amino group of glutamine and transforms into alanine by glutamic-pyruvic transaminase and α-ketoglutaric acid as by-product, which could enter the TCA cycle and transform into malic acid again. After exposure to a high level of environmental ammonia (up to 72 h), the protein and amino acid metabolism related genes in mudskippers were more down-regulated in PM than those in BP. During the treatment of 8 mM NH_4_Cl to BP and PM, mRNA levels of alanine aminotransferase and malic enzyme have been increased. Meanwhile, the mRNA levels of several important enzymes in the TCA cycle were slightly up-regulated. These data imply that partial amino acid catabolism could play an important role in reducing the production of endogenous ammonia under high environmental ammonia loading to mudskippers.

Detoxifying ammonia to urea is the main mechanism for maintaining the low internal concentration of ammonia in ureogenic and ureotelic animals. It had been suggested that mudskippers do not employ ureogenesis as the major way to cope with exogenous and endogenous ammonia during ammonia exposure [8,24]. The mRNA levels of many enzymes involved in the ornithine-urea cycle (OUC) were not up-regulated under ammonia exposure. These results, therefore, indicated that the high tolerance of mudskippers to ammonia was unrelated to urea formation and excretion.

## 4. Terrestrial Locomotion

**Figure 5:**
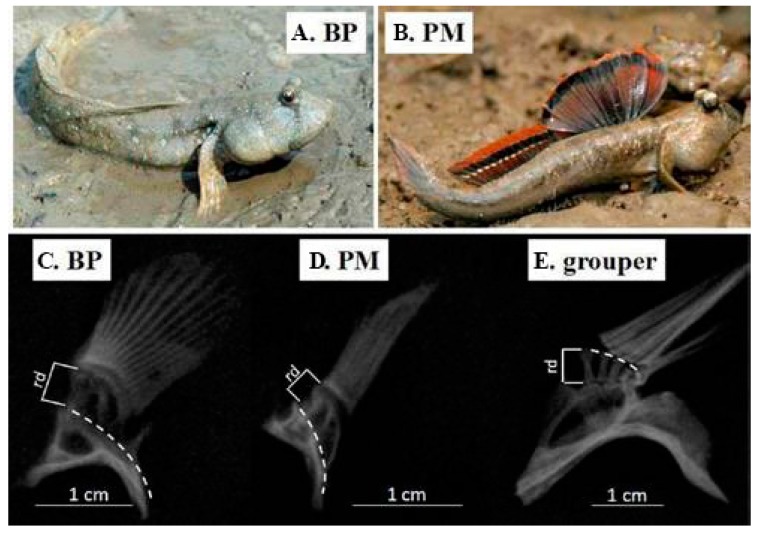
Photos of living mudskippers (**A**,**B**) and X-ray images of their pectora fins (**C**,**D**) [5,10]. A.-B. Pictures of two representative mudskipper species were taken at the field in Zhuhai of China. C.-E. X-ray images of pectoral fins, dissected from 1-year-old BP (**C**), PM (**D**) and orange-spotted grouper (**E**), were used for comparison. Dashed lines represent the body wall, and rd stands for radials.

Pectoral fins are employed for mudskippers’ locomotion on land (Figure 5A,B), while pectoral fins, the axial musculature, and caudal fins are used cooperatively for movement in water [25]. Pace and Gibb [26] assumed that the mudskipper pectoral fins may facilitate stability in water and were predisposed to be co-opted for pectoral-fin-based terrestrial locomotion. Mudskippers’ arm-like pectoral fins are attractive for scientists who study the transformation of body plans.

BP is predominantly aqueous and spends less time out of water, whereas PM is primarily terrestrial with more time on land. From the anatomical analysis on pectoral fins of BP, PM, and *Epinephelus coioides* (orange-spotted grouper; Figure 5E), we observed more protrusion of the radials in the pectoral fins of both BP and PM (Figure 5C,D), indicating the terrestrial properties of mudskippers [10]. Wang et al. [27] used an efficient high speed photography system to check the kinematics of live mudskippers and demonstrated that mudskippers used both body and pectoral fins simultaneously for locomotion in water, while they only used pectoral fins to move on land.

We also explored limb/fin-related candidate genes via scanning the available mudskipper genomes [10]. Loss of certain homeobox (*hox*) genes, elevation of Guanine-Cytosine (GC) content in a couple of *hox* clusters, and structural changes of *tbx2* and actinodin (*and*) coding genes are possible contributors to the pectoral fin protrusion in mudskippers.

**Figure 6:**
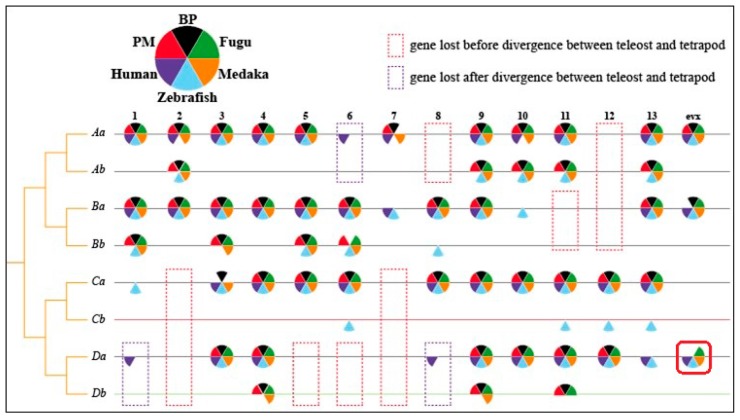
*Hox* gene clusters in fish and human [10]. The red solid box highlighted the loss of hoxDa-evx of BP and PM, which may generate neurological and morphological effects during development. Numbers 1–13 and evx are the general codes for *hox* genes.

*Hox* clusters are considered to play essential roles in encoding homeodomain-containing transcription factors for various body structures during animal development [28], and are especially critical for proper limb development where they participate in both the growth and organization of the structures [29]. Our previous works demonstrated that there are 7 clusters of *hox* genes [10] that experienced three rounds of whole genome duplication (WGD) in mudskippers (Figure 6) like other teleosts [30,31,32,33].

Interestingly, *hoxB6b* is specifically lost in the BP, and *hoxDa-evx* genes are lost both in the BP and PM (Figure 6). The movement of pectoral fins involves participation of motoneurons. Both lost *hox* genes in the two mudskippers are indirectly or directly connected to neurons. The *hoxB6a* and *hoxB6b* are recognized as the neural tube marker and rostral spinal cord marker, respectively [34], and knockout of the *hoxB6* can lead to rib and hind limb abnormality [35]. The loss of *hoxB6b* in BP may have an effect on spinal cord development and lead to the change of motor neurons in pectoral fins [10]. The *hoxD-evx* is considered as a *hox*-like gene and almost synchronously co-expressed with *hoxD13* in mammals and zebrafish [36,37]; it has also been reported to be related to the development of excitatory neurons in zebrafish [38,39]. The loss of *hoxD-evx* in BP and PM may generate neurological and morphological effects during development. Moreover, GC% of genes in *hoxAa* and *hoxBa* clusters of BP and PM are much higher than those in *hoxAb* and *hoxBb* clusters, respectively. These GC-rich *hox* clusters may play more crucial roles during embryonic development of mudskippers. Therefore, the differentiation in genomes between BP and PM might contribute to the formation of the arm-like pectoral fins [10].

Additionally, more interesting genes or proteins were analyzed to obtain more information of terrestrial locomotion. For example, T-box transcription factor 2 (*tbx2*) is considered as an important referential gene for its feature of causing limb bud outgrowth [40]. Massive and special insertions and deletions of *tbx2* were identified in the deduced protein sequence of *tbx2* in both BP and PM [10]. However, the variation of limb-specific *tbx2* could not provide convincible evidence for functional change [20]. Actinodin is a protein involved in orchestration of fin development, and it is coded from the *and* gene. Unlike other teleosts, in mudskippers the And2 protein sequences possess an alanine-rich insertion [10], which we proposed may result in an extra alpha-helix as a remarkable change.

## 5. Immunological Difference

The immune system is important to animals in anti-infection, immunological homeostasis, and immune surveillances, especially for amphibious mudskippers to live both in water and on land. Comparison of the available genome sequences among BP, PM, and other fish demonstrated that 684 gene clusters with 657 transcript-supported genes are only present in mudskippers [5]. These genes are significantly enriched in immune domains, such as Immunoglobulin-like, Immuno-globulin V-set, and Immunoglobulin subtypes.

In our previous work [5], we examined toll-like receptor 13 (*TLR13*), a family of innate immune receptors that can recognize 23S rRNA in bacteria [41]. We observed that mudskippers possess the largest number (11 copies) of *TLR13* in sequenced vertebrates so far (usually 1~3 *TLR13* in other fish). These duplicated *TLR13* and other immune-domain-containing genes may provide these amphibious fish with special immune defense, so that the mudskippers can copy with the terrestrial pathogenic microorganisms in variable (both water and terrestrial) conditions.

It is assumed that intense selective pressure acting independently on different gene families may cause the strong terrestrial adaptations for mudskippers [5]. We confirmed 722 and 705 positively selected genes (PSGs) in the genomes of BP and PM, which are markedly enriched in DNA repair, DNA replication, nucleic acid metabolism, and response to stress. These genes may contribute to maintaining genomic stability and respond to the harsh temperature gradients and direct sunlight in the intertidal zone [5,16].

## 6. Olfaction Modification

Olfactory receptor (OR) and vomeronasal system are used for olfactory sensation in mudskippers. ORs are expressed in the cell membranes of olfactory receptor neurons (ORNs) and are responsible for detection of odorants that give rise to the sense of smell. Kuciel et al. [42] provided the first comprehensive information on the immunohistochemistry and ultrastructure of the ORNs in the mudskipper *Periophthalmus barbarus* through the immunohistochemistry against OR coupled G-proteins. They observed that the ciliated ORNs were labeled by G alpha olf/s antibody, and the microvilli and crypt cells type ORNs were G alpha i-3 immunoreactive. In our previous work [5], we reported 32 and 33 ORs in BP and PM, in which 20 and 17 are aqueous feeling δ-OR, respectively. However, the number is reduced compared with most teleost (30~71). Interestingly, no air-feeling α- and γ-OR is present in mudskippers, while 200~1200 α- and γ-ORs appear in most terrestrial vertebrates. This phenomenon suggests that mudskippers have limited perception of water-borne odorants compared with other teleost, and no air-feeling OR that is required for air-borne odorant perception [5,16].

The vomeronasal system, as an accessory olfactory system in many vertebrates, could detect intraspecific pheromonal cues and some environmental odorants. Vomeronasal receptors (VR), including two subtypes of V1R and V2R, could bond with odorant molecules from air and water, respectively [43,44]. The number of *V2R* genes is more than *V1R* in mudskippers [5], in contrast with most fish. Hence, we assumed that mudskippers may use V1R for detecting air-borne chemicals on land-like tetrapods.

## 7. Air Exposure

The terrestrial properties of mudskippers are fateful for living in relative dry and hypoxic conditions on land. This phenomenon suggests that mudskippers may have special self-protective mechanisms under air exposure.

From transcriptome data (Table 1), we determined that BP and PM have 5651 and 5222 apparently up- or down-regulated genes, which indicate that mudskippers employ an energy-saving strategy associated with suppression of cell-growth and proliferation under hypoxic conditions [5]. Among the up-regulated genes, fructose- and mannose-metabolism pathway genes were significantly enriched in the liver, suggesting a potential shift towards anaerobic ATP production under hypoxia and desiccation [5,20]. Among the down-regulated genes, the gill of BP and the skin of PM possessed the relatively bigger numbers genes, suggesting that the breath of mudskipper relied more on the gill and skin.

The neurohypophysial hormones, arginine vasotocin and isotocin, regulate both hydromineral balance and social behaviors in fish. Sakamoto et al. [45] examined the effects of arginine vasotocin and isotocin administration on the amphibious behavior of individual PM in vivo. In mudskippers under terrestrial conditions, mRNA expression of brain arginine vasotocin and isotocin precursors increased 3- and 1.5-fold versus in water, respectively [45]. This may be involved in the preference for an aqueous habitat as ligands for brain isotocin receptors. The migration to land also implies the need for adaptation to dehydration. Hamasaki et al. [46] found that, when switching mudskippers from isotonic brackish water (11 psu., practical salinity unit) to seawater (ca. 34 psu) for 3 h, their body water content showed a 1% decrease compared with the ones without hypertonic challenge. They also examined vascular permeability and neuronal responsiveness to dehydration in the lamina terminalis of the mudskipper and indicated that parvocellular preoptic nucleus neurons are activated, following dehydration in mudskippers. Taken together, the vascularly permeable preoptic recess walls may be involved in osmosensing, as in the mammalian thirst center.

## 8. Conclusions

Mudskippers are capable of living out of the water, and some of their features are modified due to their amphibious way of life. In this review, we summarize the genetic features of mudskippers using genomic and transcriptomic data together with the morphological and physiological modifications of their organs. In general, these morphological and functional modifications include goggle-eyes on the head, structurally-adapted eyes with more accurate vision in air, olfactory sensation via olfactory and vomeronasal systems, special self-protective mechanisms of their skins under air exposure, stronger pectoral fins for terrestrial locomotion, specialized skin with more water holding, and aerial respiration.

On the other hand, genetic adaptations play important roles in mudskippers’ amphibious lifestyle, such as adaptive loss or mutation of certain vision-related genes. These changes could prevent UV damage to their eyes. The enzyme of AANAT for melatonin biosynthesis could mitigate the shortsightness of mudskippers on land; the loss, structural change, or specific insertion in fin-related genes may cause the pectoral fin protrusion; the duplicated *TLR13* and other immunity-domain-containing genes may generate a special immune defense; the formation of endogenous ammonia can be reduced by the active NH_3_/NH_4_^+^ excretion and low membrane permeability of ammonia in gill together with partial amino acid metabolism. These adaptations of mudskippers are quite different from those of aqueous fish; hence, they enable them to live on land.

In addition to these comparative genomic studies, we have been expanding applications to commercial and environmental conservation interests. For example, we are developing anti-bacterial peptides from the mudskipper genomes for fish food supplements [47]. We are also propagating these amphibious fish for their important roles in environmental preservation. Recently, numerous mudskippers have been released into wetland parks along the coast of Shenzhen Bay (next to Hong Kong), aiming to restore the mudskipper population and attract birds (which feed on the fish) for the improvement of suburban conditions in this modernized city in the Southern China.

The attractive terrestrial features provide a good opportunity to study the transitional evolution from aqueous to terrestrial conditions. In our previous studies, we provided a new model to understand the adaptive evolution from water to land. An analysis of the mudskippers’ genome and transcriptome sequences will also provide insights into the fields of molecular breeding, functional identification, natural products, anti-bacterial peptides, aqueous feed additives, and environmental preservation, and may stimulate further theoretical considerations.

## Figures and Tables

**Table 1:** Up- and down-regulated gene numbers in BP and PM under air exposure (modified from [5]).

Tissue	BP		PM	
	Up-Regulated	Down-Regulated	Up-Regulated	Down-Regulated
Brain	117	177	519	409
Gill	192	1435	682	730
Liver	1353	638	553	553
Muscle	580	1448	429	124
Skin	175	272	487	1510
Total	2207	3444	2305	2917
